# Editorial: Role of endophytic bacteria in improving plant stress resistance

**DOI:** 10.3389/fpls.2022.1106701

**Published:** 2022-12-06

**Authors:** Yang Liu, Massimiliano Morelli, Janne J. Koskimäki, Sheng Qin, Yong-Hua Zhu, Xiao-Xia Zhang

**Affiliations:** ^1^ School of Chemistry and Biological Engineering, University of Science and Technology Beijing, Beijing, China; ^2^ Consiglio Nazionale delle Ricerche, Istituto per la Protezione Sostenibile delle Piante, Sede Secondaria di Bari, Bari, Italy; ^3^ Ecology and Genetics Research Unit, University of Oulu, Oulu, Finland; ^4^ The Key Laboratory of Biotechnology for Medicinal Plant of Jiangsu Province, School of Life Sciences, Jiangsu Normal University, Xuzhou, China; ^5^ Hunan Province Key Laboratory of Plant Functional Genomics and Developmental Regulation, College of Biology, Hunan University, Changsha, Hunan, China; ^6^ Institute of Agricultural Resources and Regional Planning, Chinese Academy of Agricultural Sciences, Beijing, China

**Keywords:** endophytic bacteria, plant stress resistance, plant growth-promoting bacteria, plant-microbe interaction, holobiont, omics

Endophytic bacteria inhabiting plant tissues are generally considered to play a crucial role in host adaptation to biotic stresses and adverse environmental conditions (Compant et al., 2021; Oukala et al., 2021). In recent years, the interest in endosphere-associated microbes has grown exponentially (Morelli et al., 2020). A deeper understanding of the biology of bacterial endophytic communities and their intimate relationships with the plant genetic network, is paving the way for advancing knowledge on the microbial impact on the plant stress response, tolerance, and adaptation (Tamosiune et al., 2017). Researchers have recognized that a thorough examination of the interplay between endophytes and their host plants may hold the key to developing multi-factor control strategies for the most common stress drivers affecting plants, especially in unfavorable environments. This novel approach also provides a better perspective to study the potential of endophyte-related biological activities, such as biocontrol, bioremediation, and metabolite production (Pirttilä et al., 2021).

This Research Topic aims to present new research results focusing on the role of endophytic bacteria and the complex interactions they establish with plants to improve host resistance to stress under different environmental conditions.

Endophytic bacteria produce diverse metabolites that may benefit the plant hosts in the growth promotion and control of diseases (Compant et al., 2013). Many different modes of action have been described to explain how endophytic bacteria enhance the ability of plants to withstand biotic adversities (Ab Rahman et al., 2018). In some cases, they could compete for nutrients with pathogens within colonized tissues, secrete antibiotic molecules and antimicrobial peptides, or even trigger plant defence responses and induced systemic resistance (ISR) (Dini-Andreote, 2020). Mutungi et al. showed colonization of common bean (*Phaseolus vulgaris*) by endophytic strains of *Bacillus megaterium* and *Enterobacter hormaechei*, respectively isolated from the perennial herbs *Boeravia erecta* and *Abutilon fruticosum*, promoted plant growth and reduced the severity of the disease caused by infection with *Fusarium solani*. The growth inhibition of *F. solani* was also confirmed by co-culturing with these endophytes *in vitro*. Both strains possessed traditional plant growth-promoting traits for the production of phytohormone indole acetic acid (IAA) and solubilization of phosphates. Similarly, Gupta et al. reported that endophytic strains of *Pseudomonas aeruginosa* and *Aneurinibacillus aneurinilyticus*, isolated from “*tulsi*” (*Ocimum tenuiflorum*) plants, could induce ISR in pea (*Pisum sativum*) when challenged with pathogenic Fusarium wilt (*Fusarium oxysporum* f.sp. *pisi*). It was hypothesized that these endophytes could exert their antagonistic and growth-promoting effects by releasing secondary metabolites, including volatile organic compounds (VOCs), siderophores, and ammonia, or by mobilizing hitherto inaccessible inorganic nutrients.

Interestingly, the involvement of endophytic traits for phosphate solubilization, iron acquisition, ACC deamination, nitrogen fixation, and IAA production was shown to underpin the mechanism used by *Streptomyces lincolnensis*, isolated from wormwood (*Artemisia annua*), to regulate the expression of genes controlling root morphogenesis in *Arabidopsis thaliana* (Fu et al.).

Controlling the uptake and bioavailability of micronutrient, mineral, and trace element levels in plants plays an essential role in plant growth and development. Fine regulation of these steps also involves interaction between the plant host and its microbial colonizers (D’Attoma et al., 2019). Moreover, mobilization of nutrients, nitrogen fixation, and siderophore-mediated iron capture are also the mechanisms most commonly deployed by endophytes to impart tolerance to abiotic stress in plants facing harsh environmental conditions (Truyens et al., 2015; Abideen et al., 2022; Liu et al., 2022).

The ability to produce antimicrobial peptides is an additional feature of interest in bacterial endophytes (Singh et al., 2017). Edeines are non-ribosomal peptides with significant antibacterial effects produced by *Brevibacillus brevis* (Johnson et al., 2020). Du et al. investigated the molecular basis of edeine production in *B. brevis*, identifying in EdeB a regulatory factor that significantly increases peptide release.

The high-resolution detection power of omics approaches can be used to unravel the complex role of endophytic communities, along with the dynamics of their interactions with host plants (Lucaciu et al., 2019; Agrahari et al., 2020; Anguita-Maeso et al., 2020; Giampetruzzi et al., 2020). Similar culture-independent omics approaches have been successfully applied to the computational analysis of the complex network of interaction between plants and root-associated microorganisms inhabiting the rhizosphere (White et al., 2017).

Gu et al. established new computational tools for ecological network analysis of high-throughput sequencing data. The study revealed that the continuous cropping regime significantly alters the composition and dynamics of the belowground microbiomes associated with different potato cultivars.

Chen et al. utilized high-throughput sequencing to screen bacterial communities in farmlands of South China and investigate whether multiple cropping farm systems alter the stability of the soil bacterial community structures. They discovered that tobacco-rice crop rotation increased the richness of microbial diversity, with potential benefits for soil properties and crop yields. The study provides insights for ensuring soil quality and enhancing sustainable agricultural practices and production capacity.

Chen et al. used 16S rRNA high-throughput sequencing of peanut (*Arachis hypogaea*) rhizosphere to understand whether neighboring maize crops could alter root microbiome assembly. They found that maize coexistence re-shaped peanut-associated belowground microbial communities and induced colonization by plant-growth-promoting rhizobacteria, such as *Bradyrhizobium* sp. and *Streptomyces* sp., with relevant potential for effective nutrient accumulation and phytohormone production.

Similarly, Zhang et al. (2022) showed that maize could selectively shape its internal microbial community by recruiting bacterial endophytes carrying nitrogenase genes (*nifH*) to enhance nitrogen fixation. Taken together, these findings reinforce the hypothesis that plants establish a plant-microbial holobiont through a selective co-evolution with their endophytic inhabitants (Fagorzi and Mengoni, 2022) and this, in turn, improves plant fitness and resistance to adverse stimuli.

Modern sustainable agriculture is nowadays looking at this “plant holobiont breeding” approach (Wei and Jousset, 2017) which attempts to stimulate plant-microbial symbiosis by cultivating certain crops while considering the fluctuating interaction with their endophytes, and thus selecting different host genotypes based on their associated microbial communities (Sahu and Mishra, 2021). Future plant breeding should take microbial elements into account since changing the endophyte species introduced from the environment might change the host plant’s genetic background, influencing the resulting phenotypic traits. Gaining knowledge of endophytic communities interacting with newly introduced crop varieties, and attempting their re-shaping, based on effects on plant phenotypic and developmental traits, may represent an unprecedented resource to be exploited to increase crop productivity (Mukherjee et al., 2021).

Bringing together seven original research papers, this Research Topic provides a state-of-the-art overview of the beneficial effect that bacterial endophytes may exert on plants from different perspectives, with special emphasis on their ability to increase the potential of host plants to tolerate biotic and abiotic stresses.

We regard this collective effort as an important opportunity to recall the importance of directing future research on plant endophytes towards an in-depth analysis of their modes of interaction with hosts. Based on this approach, rapid progress could be made in the discovery of natural plant growth promoters and microbial fertilizers to provide sustainable alternatives to the massive use of synthetic pesticides, hindered by the “Green Deal” innovations (Tataridas et al., 2022). We hope that this Research Topic will provide some up-to-date references to achieve this aim.

## Author contributions

YL and MM wrote the first draft of the manuscript. X-XZ, JK, SQ and Y-HZ edited the manuscript and provided a critical review. X-XZ submitted the final version. All authors made a substantial, direct and intellectual contribution to the work, and approved the submitted version.
